# Small RNA inhibits infection by downy mildew pathogen *Hyaloperonospora arabidopsidis*

**DOI:** 10.1111/mpp.12863

**Published:** 2019-09-26

**Authors:** Özlem Bilir, Osman Telli, Chris Norman, Hikmet Budak, Yiguo Hong, Mahmut Tör

**Affiliations:** ^1^ Department of Biology School of Science and the Environment University of Worcester Henwick Grove Worcester WR2 6AJ UK; ^2^ Montana BioAgriculture Inc. Missoula Montana USA; ^3^ Research Centre for Plant RNA Signaling College of Life and Environmental Sciences Hangzhou Normal University Hangzhou 310036 China; ^4^ Present address: Directorate of Trakya Agricultural Research Institute Department of Biotechnology D‐100 Highway 22100 Edirne Turkey

**Keywords:** *Arabidopsis*, downy mildew, oomycetes, spray‐induced gene silencing (SIGS), sRNA

## Abstract

Gene silencing exists in eukaryotic organisms as a conserved regulation of the gene expression mechanism. In general, small RNAs (sRNAs) are produced within the eukaryotic cells and incorporated into an RNA‐induced silencing complex (RISC) within cells. However, exogenous sRNAs, once delivered into cells, can also silence target genes via the same RISC. Here, we explored this concept by targeting the *Cellulose synthase A3 (CesA3)* gene of *Hyaloperonospora arabidopsidis* (*Hpa*), the downy mildew pathogen of *Arabidopsis thaliana. Hpa* spore suspensions were mixed with sense or antisense sRNAs and inoculated onto susceptible *Arabidopsis* seedlings. While sense sRNAs had no obvious effect on *Hpa* pathogenicity, antisense sRNAs inhibited spore germination and hence infection. Such inhibition of infection was not race‐specific, but dependent on the length and capping of sRNAs. Inhibition of infection by double stranded sRNA was more efficient than that observed with antisense sRNA. Thus, exogenous sRNA targeting conserved *CesA3* could suppress *Hpa* infection in *Arabidopsis*, indicating the potential of this simple and efficient sRNA‐based approach for deciphering gene functions in obligate biotrophic pathogens as well as for *R*‐gene independent control of diseases in plants.

## Introduction

Noncoding 20–30 nucleotide (nt)‐long small RNAs (sRNAs) have been known to be involved in the regulation of gene expression and defence in eukaryotes (Chen *et al.*, 2018; Qin *et al.*, 2017; Zhang *et al*., 2019). Different types of RNAs, such as double‐stranded RNA (dsRNA) and small interfering RNA (siRNA), can trigger homologous RNA degradation or inhibit mRNA translation (Huang *et al.*, 2016; Nejat and Mantri, 2018). This process is known as RNA silencing and plays a significant role in various biological processes, including innate immunity (Brant and Budak, 2018; Deng *et al.*, 2018) and development (Li *et al.*, 2017; Qin *et al.*, 2017).

In plant–microbe interactions, plants and microbes can exchange RNA molecules, which then integrate into RNA silencing machinery in reciprocal recipient cells. Such cross‐kingdom RNA transfer was first demonstrated between fungus and plants (Weiberg *et al.*, 2013). *Botrytis cinerea*, an ascomycete fungus infecting more than 200 plant species, transports its sRNAs that silence both *Arabidopsis* and tomato genes by hijacking plant cellular gene silencing machinery (Weiberg *et al.*, 2013). On the other hand, a large number of cotton sRNAs were found in *Verticillium dahliae* hyphae recovered from *V. dahliae-*infected plant tissues and some of the cotton‐originated sRNAs can target essential fungal virulence genes (Zhang *et al.*, 2016).

Movement of sRNAs from plant to pathogens has been explored using the host‐induced gene silencing (HIGS) technique where the sRNAs are generally made by producing dsRNA in transgenic plants using *Agrobacterium* or in viruses that replicate through dsRNA. HIGS has been successfully used to suppress essential pathogen genes in various plant–pathogen interaction systems including barley–*Fusarium* (Koch *et al.*, 2013), *Arabidopsis–* and tomato–*Verticillium* (Song and Thomma, 2018), barley– and wheat–*Blumeria* (Nowara *et al.*, 2010) and lettuce–*Bremia* (Govindarajulu *et al.*, 2015). Similarly, HIGS has also been used against nematodes in *Arabidopsis* (Huang *et al.*, 2006).

Several recent studies have shown that exogenously applied sRNAs can be taken up by fungal or plant cells and trigger RNA silencing. The exogenous application of RNA by spraying it directly onto plants has been referred to as spray‐induced gene silencing (SIGS) (Koch *et al.*, 2016). The induction of gene silencing by spraying, or otherwise applying RNA, avoids the need to develop transgenic plants (Wang and Jin, 2017). This method has been tested against fungal pathogens, including *Fusarium graminearum* (Koch *et al.*, 2016) and *Fusarium culmorum* (Koch *et al.*, 2018). Several different methods have been used to deliver sRNAs onto plants. For example, high‐pressure spraying siRNAs was found to efficiently silence transgenic GFP gene expression in *Nicotiana tabacum* (Dalakouras *et al.*, 2016). In a different approach, Mitter *et al. *(2017) used clay nanosheets to deliver dsRNA onto plants for silencing homologous viral RNA. For insect pests, an ingestion method seems to be another way to deliver RNAs. Insects were fed an artificial diet containing dsRNAs in order to induce RNA silencing. This strategy was successfully exploited to target against coleopteran species such as western corn rootworm *Diabrotica virgifera virgifera* (Baum *et al.*, 2007).

The oomycetes include a unique group of biotrophic and hemibiotrophic plant pathogens and are distinct from fungi (Kamoun *et al.*, 2014). The cell walls of oomycetes have been reported to be primarily β‐1,3‐glucans and cellulose with little or no chitin (Kamoun, 2003). Oomycete hyphae are coenocytic (multinucleate with no division by septa) and their vegetative nuclei are in a diploid state (Fugelstad, 2008; Coates and Beynon, 2010). Genetic manipulation has been developed for some oomycete pathogens. For example, stable transformations using protoplast uptake and regeneration have been reported for the culturable oomycetes including *Phytophthora infestans*, *Phytophthora sojae* and *Phytophthora citricola* (Kamoun, 2003; Mcleod *et al.*, 2008). Using a DNA‐directed RNAi system, efficient gene silencing has been achieved in *P. infestans* (Abrahamian *et al.*, 2016). Saraiva *et al. *(2014) used uptake of dsRNA into protoplasts of *Saprolegnia parasitica* and reported the efficient silencing of the tyrosinase gene. However, routine genetic transformations or gene silencing studies in obligate oomycete species have been hampered by the lack of efficient reliable methods.

*Hyaloperonospora arabidopsidis* (*Hpa*) is an obligate biotrophic oomycete pathogen that causes downy mildew disease on *Arabidopsis thaliana.* The *Hpa–Arabidopsis* system has been used as a model to investigate pathogen effectors (Woods‐Tör *et al.*, 2018) and plant immunity (Holub, 2007). This pathogen has both sexual and asexual reproduction. For infection, an asexual conidiospore germinates on the surface of plant leaves, forms an appressorium, then a penetration hypha grows between the walls of neighbouring epidermal cells (Koch and Slusarenko, 1990). In susceptible plants, the hypha branches out into the intercellular space and forms haustoria in the epidermal and mesophyll cells. Within 1–2 weeks, conidiophores develop through stomata, carrying conidiospores that begin new rounds of infection. Sexual spores, called oospores, are produced within the cotyledons or on the leaves of the infected plant (Koch and Slusarenko, 1990).

To our knowledge, there is no reliable and efficient genetic transformation method for *Hpa*. Here, we used *in vitro* synthesized sRNAs targeting the *Hpa‐CesA3* gene and report that antisense or double‐stranded capped sRNAs of 25 nt or longer inhibit spore germination and hence infection.

## Results

### Selection of target gene for sRNA‐mediated silencing in *Hpa*

**Figure 1:**
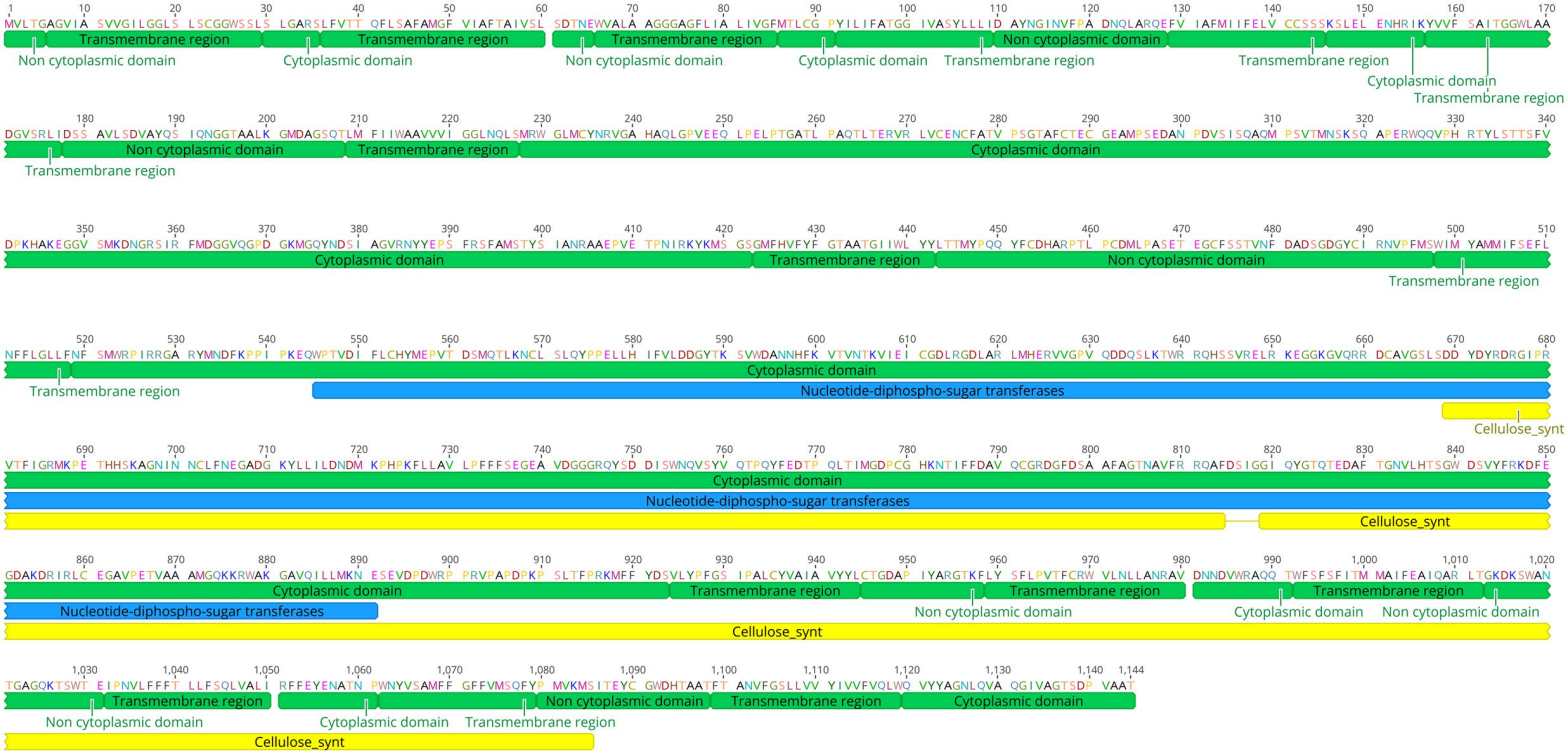
Domain structure of *Hpa*‐CesA3. The mature protein has 15 transmembrane domains, a nucleotide‐diphospho‐sugar transferase domain (amino acid residues 545‐891) and a cellulose synthase domain (amino acid residues 814‐1122). sRNA is the region where sRNAs were designed.

The main cell wall components of oomycetes are β‐glucans and cellulose (Fugelstad, 2008; Raaymakers and Van den Ackerveken, 2016). We focused on a cellulose synthase gene as a target for sRNA‐mediated silencing. Using Pfam (Punta *et al.*, 2012), we identified M4BU64 of *Hpa* belonging to the cellulose synthase gene family. *In silico* analysis revealed that this gene corresponds to *HpaG810051* in the Emoy2 genome and exists as a single copy gene. EnsemblProtists gene annotation revealed *HpaG810051* does not have an intron and the open reading frame encodes a predicted protein of 1144 amino acids (molecular mass 127.028 kDa). A BLASTX search against the database revealed that HpaG810051 has a high similarity to CesA3 proteins from other oomycetes, thus we designated *HpaG810051* as *Hpa‐CesA3*. We then obtained the nucleotide and amino acid sequences of the *CesA3* genes of *Albugo candida*, *Albugo laibachii*, *Bremia lactucae*, *Phytopthora capsici*, *P. infestans* and *Plasmopara viticola* and aligned them with those of *Hpa*‐*CesA3*. Alignment of amino acid sequences revealed a 93% identity of *Hpa*‐CesA3 with *P. capsici*‐CesA3, 92% with *P. infestans*‐CesA3 and *P. viticola*‐CesA3, 91% with *B. lactucae*‐CesA3, 77% with *A. candida*‐CesA3 and 76% with *A. laibachii*‐CesA3 (Fig. S1). Domain and motif searches of the *Hpa‐*CesA3 revealed a nucleotide‐diphospho‐sugar transferase domain (W545‐E891) and a cellulose synthase domain (F814‐Y1122), as well as 15 transmembrane domains (Fig. 1). Alignment of nucleotide sequences of these seven genes showed that *Hpa‐CesA3* had 65–84% identity to its orthologues (Fig. S2). Interestingly, nucleotide alignment showed no region with 100% identity that would allow designing of a common sRNA for *CesA3* gene silencing across these oomycete species.

We then looked at the expression pattern of *Hpa‐CesA3* using the available published transcriptome data in *Arabidopsis* Col‐0 inoculated with the avirulent or virulent *Hpa* isolates Emoy2 or Waco9, respectively (Asai *et al*., 2018). It is clear that *Hpa‐CesA3* is expressed highly in the spores and the level of expression drops significantly in the mycelia during development (Fig. S3).

### *Hpa‐CesA3* antisense sRNA inhibits *Hpa* sporulation

**Figure 2:**
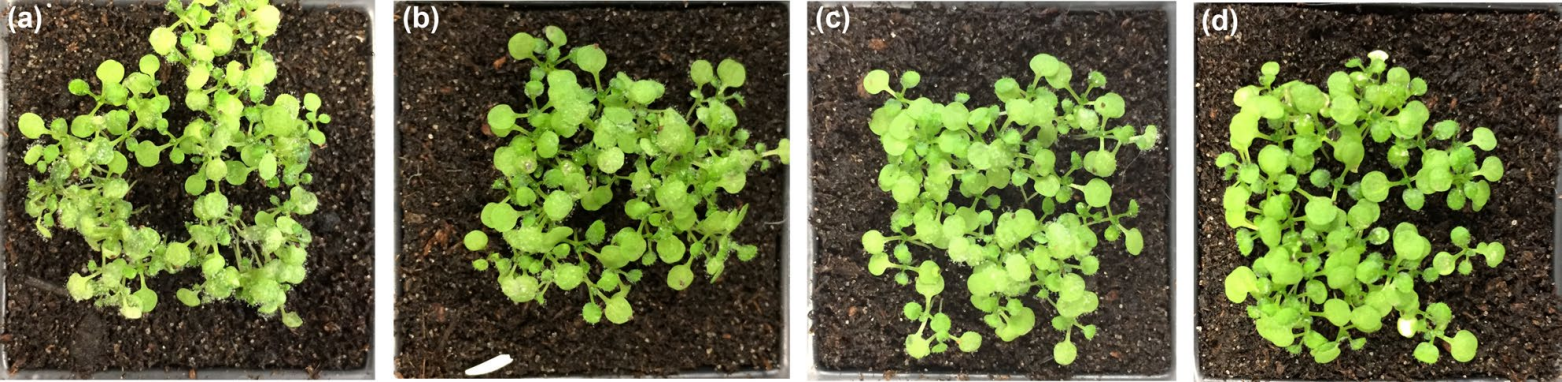
Application of antisense sRNA targeting *Hpa‐CesA3* inhibits sporulation. *Hpa*‐Emoy2 spores were mixed with antisense sRNA at different concentrations and 7‐day‐old *Arabidopsis* seedlings were drop inoculated. Seedlings were examined for sporulation at 7 days post‐inoculation. (a) Control (no antisense sRNA), (b) seedlings inoculated with spores mixed with 5 µM antisense sRNA, (c) seedlings inoculated with spores mixed with 10 µM antisense sRNA and (d) seedlings inoculated with spores mixed with 20 µM antisense sRNA. The inoculation experiments were repeated five times and similar observations were made. There was no sporulation in seedlings inoculated with spores mixed with 20 µM antisense sRNA.

**Figure 3:**
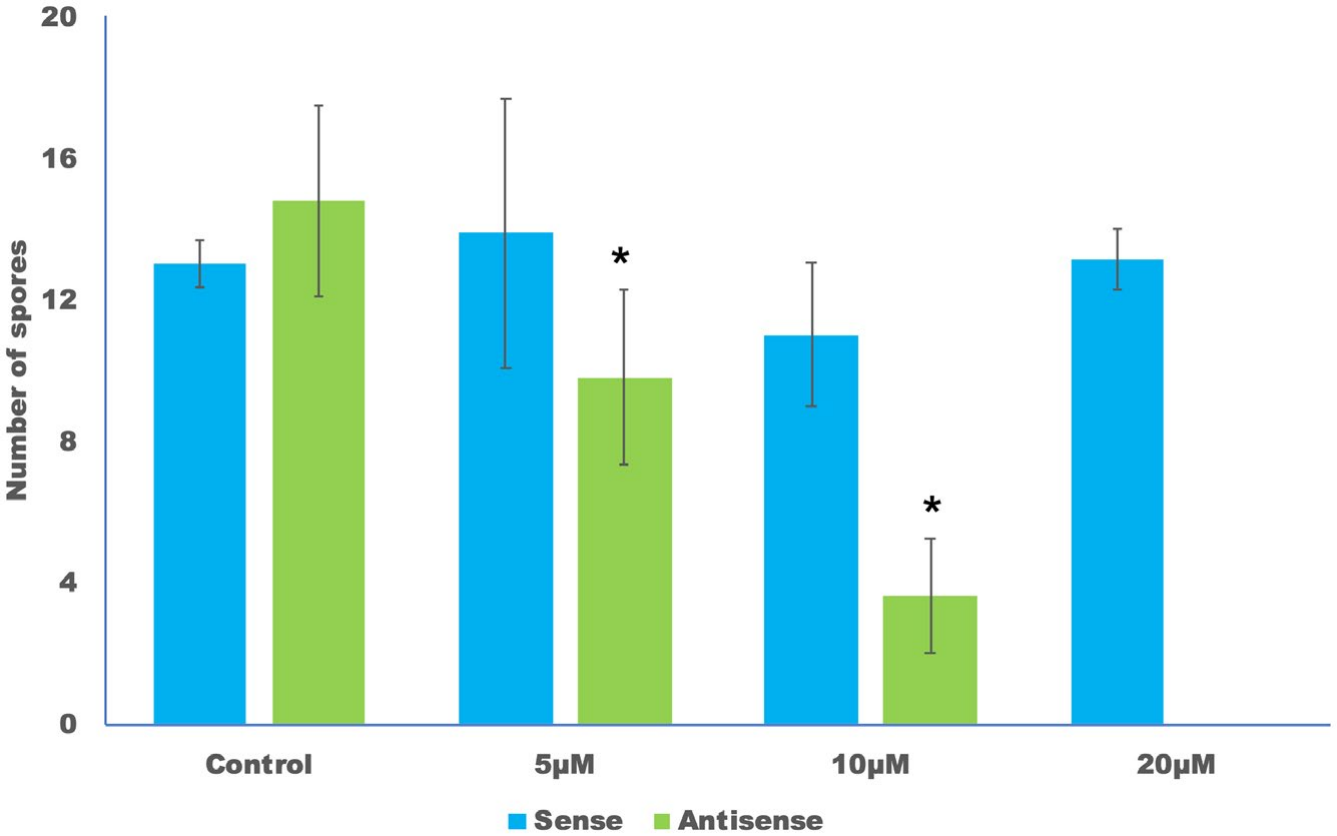
Antisense but not sense sRNA inhibits sporulation. *Arabidopsis* seedlings were drop inoculated with *Hpa*‐Emoy2 spores containing 0, 5, 10 and 20 µM sense or antisense sRNA. Ten inoculated seedlings from each sample were collected at 7 days post‐inoculation and placed in 250 µL H_2_O. The number of spores was counted using a haemocytometer. The average and standard error of three replicates are shown. The experiment was repeated five times with similar results. Asterisks (*) indicate significant difference to control inoculation at the corresponding sRNA (*P* < 0.05, paired Student’s *t*‐tests).

Since *Hpa* is an obligate biotrophic pathogen and grows on *Arabidopsis*, we checked whether *Hpa‐CesA3* had any homology to *Arabidopsis CesA* genes (Burn *et al.*, 2002). BLASTN searches against the *Arabidopsis* database revealed no significant similarity. Subsequently, we designed 25‐nt sense and antisense RNA oligonucleotides from the 5ʹ region of the gene that does not have any homology in other genes in the *Hpa* genome. The sense or antisense sRNAs were mixed with *Hpa* spores at 5, 10 and 20 µM concentrations and 7‐day‐old *Arabidopsis* seedlings were drop inoculated. At 7 days post‐inoculation (dpi), *Hpa* sporulation was checked and no visible difference in sporulation was observed between control plants (Fig. 2a) and those inoculated with spore suspensions containing 5, 10 or 20 µM sense sRNA. However, sporulation was visibly reduced on plants inoculated with spore suspensions containing 5 and 10 µM antisense RNA (Fig. 2b,c). Interestingly, there was no sporulation on plants inoculated with a spore suspension containing 20 µM antisense RNA (Fig. 2d). Quantitative data analysis further demonstrated the significant reduction or no sporulation in plants inoculated with *Hpa* spores mixed with antisense sRNAs (Fig. 3). The experiment was repeated at least five times, each time with a minimum of three replicates, and similar results were obtained. We also designed 25‐nt sense and antisense DNA oligonucleotides from the same region of the gene and carried out similar inoculation experiments with 20 µM DNA oligonucleotides. There was no difference in the sporulation between control plants and those inoculated with sense or antisense DNA oligonucleotides (Fig. S4).

### *Hpa‐CesA3* antisense sRNA inhibits spore germination

**Figure 4:**
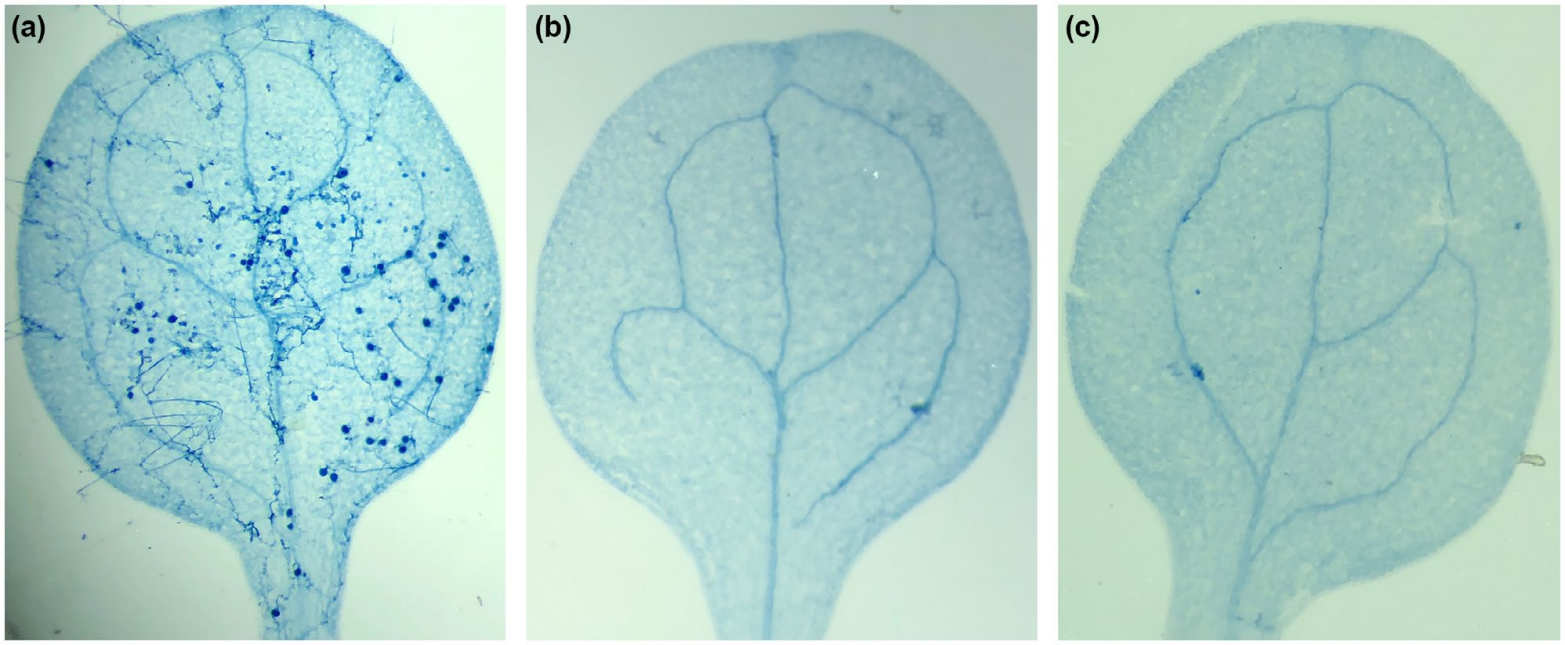
Pathogen inhibition at the infection stage. *Arabidopsis* seedlings were drop inoculated with *Hpa*‐Emoy2 spores containing 0 or 20 µM antisense sRNA and at 7 days post‐inoculation seedlings were stained with trypan blue. While there was normal infection with sporulation and oospore development was observed in the control (a), no infection or hyphal development were observed in seedlings inoculated with 20 µM antisense sRNA (b) and (c).

**Figure 5:**
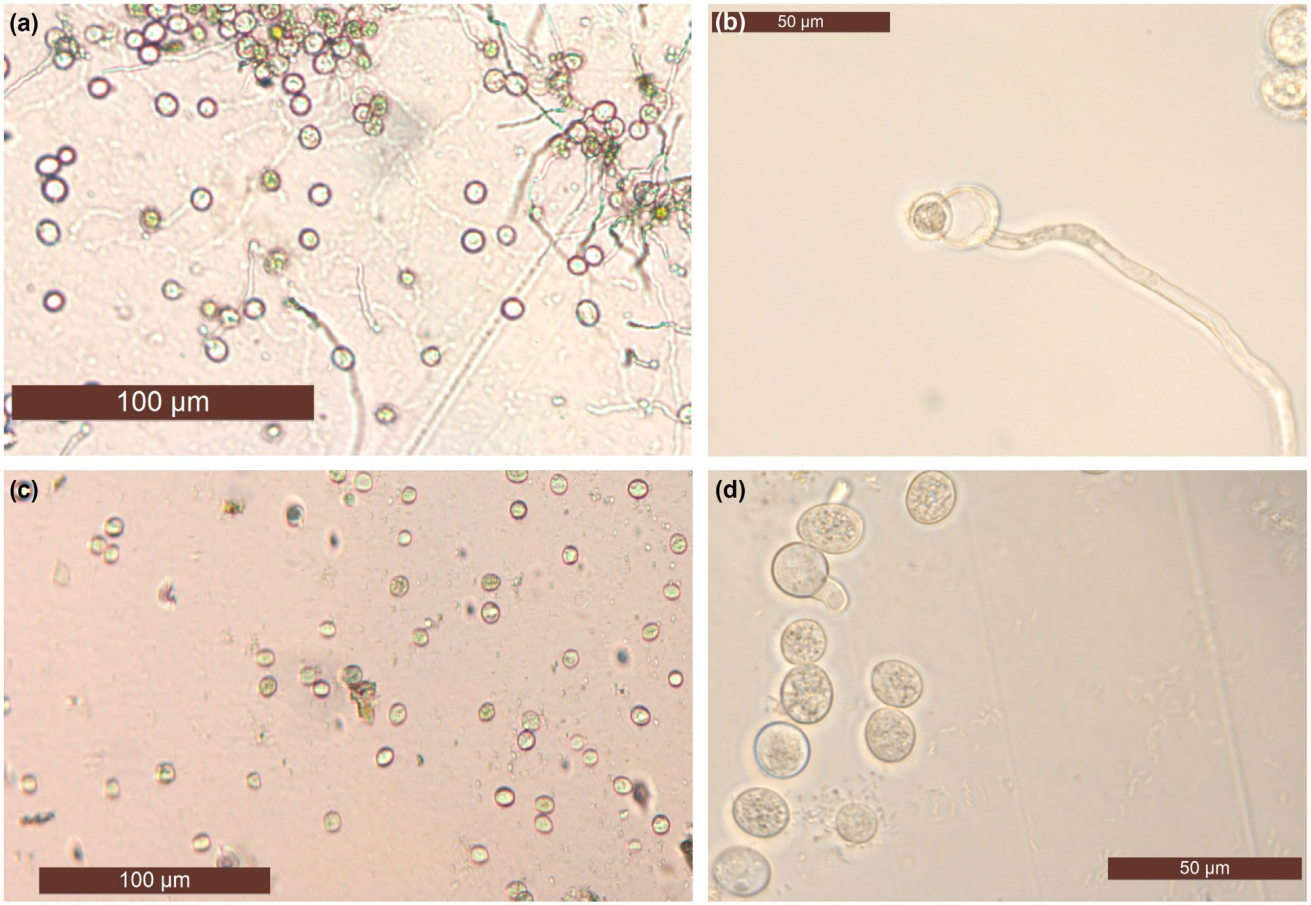
Germination is inhibited by antisense sRNA targeting the *Hpa‐CesA3* gene. *Hpa*‐Emoy2 spores containing 0 or 20 µM antisense sRNA were placed on cellophane strips on MS medium and spore germination was examined using a Leica DM5500B light microscope after 48 h. Controls (a) and (b) produced long germination tubes, while spores incubated with 20 µM antisense sRNA (c) did not germinate or the germination tube was arrested (d) within the given time period.

To investigate how *Hpa‐CesA3* antisense sRNA inhibits sporulation, we inoculated 7‐day‐old *Arabidopsis* seedlings with a spore suspension containing 20 µM antisense sRNA. Trypan blue staining of inoculated leaves was carried out at 7 dpi to reveal the extent of *Hpa* development in the tissues. Although normal pathogen development was observed in the control leaf tissues (Fig. 4a), there were no hyphae in, or pathogen spores on, the cotyledons inoculated with the spore suspension containing 20 µM sRNA (Fig. 4b,c). This indicates that antisense sRNA may have inhibited spore germination, thus preventing infection. Non‐germinating spores may have been washed away during trypan blue staining. To investigate this further, we set up germination assays using cellophane strips. After 48 h spores were examined for germination under a light microscope. Untreated, control spores were bright and produced germ tubes at various lengths within the 2 days (Fig. 5a,b). However, spores treated with antisense sRNA became dark brown and germination tubes were mainly absent or, in rare cases, were arrested (Fig. 5c,d). We repeated this assay five times and observed 100% inhibition of germination in all experiments (Table S1).

### *Hpa‐CesA3 *antisense sRNA‐mediated suppression of *Hpa* infection is not race specific

*Hpa‐CesA3* antisense sRNA‐mediated suppression of *Hpa* infection was carried out using the *Hpa*‐Emoy2 isolate. To determine whether or not the suppression of infection we observed was isolate‐specific, we carried out a similar study using *Hpa*‐Cala2 isolate. Using BLASTN, we determined that the Emoy2 *Hpa‐CesA3* gene had 99.97% identity to that of Cala2 and 100% identity at the sRNA target region (Fig. S5). Subsequently, we inoculated 7‐day‐old *Arabidopsis* seedlings with *Hpa*‐Cala2 spores mixed with and without 20 µM antisense sRNA. As expected, we observed normal sporulation in control seedlings while seedlings inoculated with spore suspension containing 20 µM antisense sRNA did not show any sporulation (data not shown), indicating *Hpa‐CesA3* antisense sRNA‐mediated suppression of *Hpa* infection was not race‐specific.

### Capping antisense sRNA is essential for suppression of *Hpa* infection

**Figure 6:**
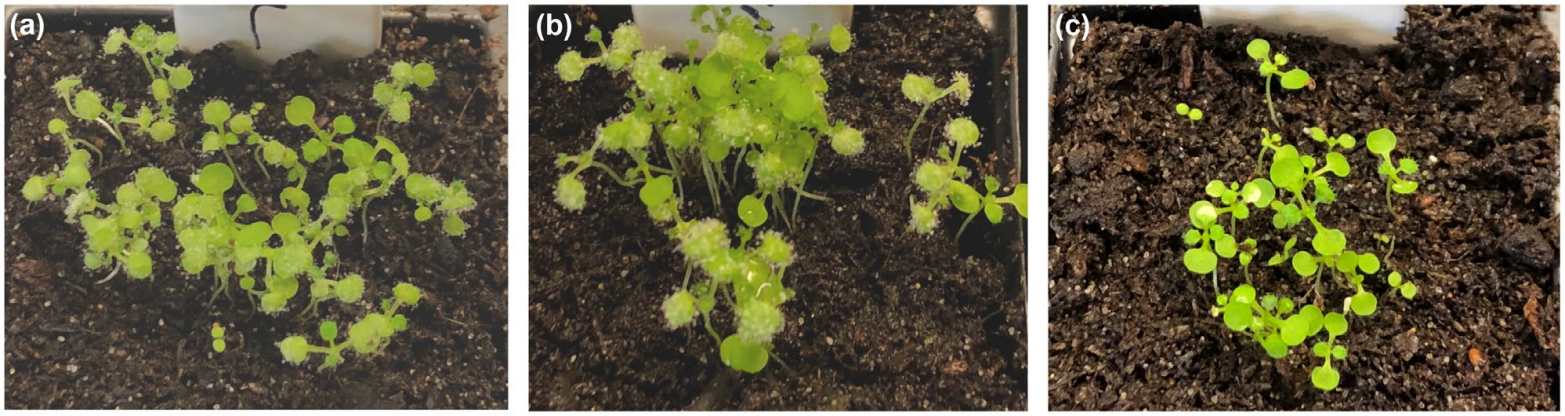
Capping sRNA has an effect on gene silencing. *Arabidopsis* seedlings were inoculated with *Hpa*‐Emoy2 spore suspension containing no sRNA (a), 20 µM uncapped antisense sRNA (b) and 20 µM capped antisense sRNA (c). Experiments were repeated five times with similar results. Samples were photographed at 7 days post‐inoculation.

The experiments described above were carried out with 25 nt capped antisense sRNAs. To determine whether capping influenced the silencing of *Hpa‐*CesA3, we obtained an uncapped version of the same antisense sRNA and carried out similar inoculation studies. After 7 dpi, the control *Arabidopsis* seedlings and those seedlings inoculated with spores mixed with uncapped antisense 25 nt sRNA developed typical *Hpa* infection, resulting from normal sporulation and germination (Fig. 6a,b). In the same experiments, inoculations with spores mixed with capped antisense sRNA showed neither germination nor sporulation (Fig. 6c). These results reveal that sRNA capping is essential for sRNA biological activity in suppressing *Hpa* infection of plants.

### sRNA length has an impact on *Hpa‐CesA3 *antisense sRNA‐mediated suppression of *Hpa* infection

**Figure 7:**
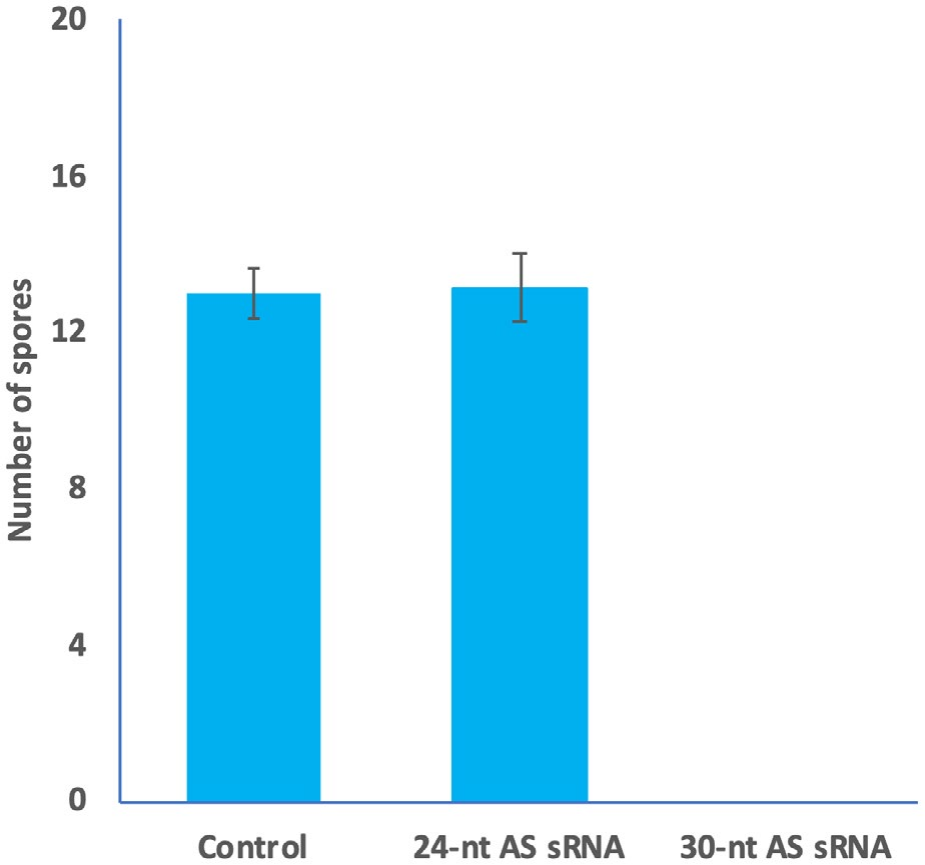
Length of sRNA has an effect on pathogen development. *Arabidopsis* seedlings were drop inoculated with *Hpa*‐Emoy2 spore suspension containing no sRNAs (control), 24 nt antisense (AS) sRNA and 30 nt antisense sRNAs. The inoculated seedlings from each sample were collected at 7 days post‐inoculation and placed in 250 µL H_2_O. The number of spores was counted using a haemocytometer. No spores were detected in samples inoculated with spore suspension containing 30 nt antisense sRNA. The average and standard error of three replicates are shown. The experiment was repeated five times with similar results.

The length of sRNA could influence the gene silencing (Vargason *et al.*, 2003). In addition to the 25 nt sRNA, we tested the effect of 24 and 30 nt antisense *Hpa‐CesA3* sRNAs on *Hpa* infection. Using 24 nt sRNAs, normal sporulation was observed on the seedlings inoculated with 20 µM sRNAs and plants became infected with *Hpa*. However, there was no sporulation on seedlings inoculated with spores containing 20 µM 30 nt antisense sRNAs and the inoculated *Arabidopsis* plants remained healthy (Fig. 7).

### Double‐stranded sRNA is more effective than single‐stranded sRNA in inhibiting infection

**Figure 8:**
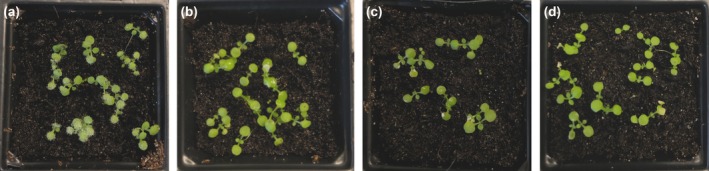
Double‐stranded (ds) sRNA is more effective in inhibiting infection than single‐stranded sRNA. *Hpa*‐Emoy2 spores were mixed with ds sRNA at 0, 5, 10 and 20 µM concentrations and 7‐day‐old *Arabidopsis* seedlings were drop inoculated. Seedlings were examined for sporulation at 7 days post‐inoculation. (a) Control (no sRNA), (b) seedlings inoculated with spores mixed with 5 µM ds sRNA, (c) seedlings inoculated with spores mixed with 10 µM ds sRNA and (d) seedlings inoculated with spores mixed with 20 µM ds sRNA. The inoculation experiments were repeated five times and similar observations were made. There was no sporulation in seedlings inoculated with spores mixed with 5, 10 or 20 µM ds sRNA.

Since application of 5 and 10 µM antisense sRNA allowed reduced sporulation in infected plants (Fig. 2), we investigated whether double‐stranded (ds) sRNA would work better than the single‐stranded sRNA in inhibiting infection. We drop inoculated *Arabidopsis* seedlings with spores mixed with 0, 5, 10 and 20 µM 30‐nt dsRNA. At 7 dpi, *Hpa* sporulation was checked and while normal sporulation was observed in control seedlings, no sporulation was detected on seedlings inoculated with spore suspension containing 5, 10 and 20 µM 30‐nt dsRNA (Fig. 8). The experiment was repeated three times, each time with a minimum of three replicates, and similar results were obtained. Quantitative data analysis supported our observation (Table S2).

## Discussion

Using the *Arabidopsis–Hyaloperonospora* model system, we showed that targeting the *Hpa‐CesA3* gene in Emoy2 and Cala2 isolates by exogenously applying gene‐specific sRNAs inhibits germination and hence infection of *Arabidopsis*.

We chose the *Hpa*‐*CesA3* gene because cellulose is an important structural component of the cell wall of oomycetes (Raaymakers and Van den Ackerveken, 2016). Blum *et al. *(2012) investigated *CesA3* genes in a total of 25 different oomycete species originating from the six ‘crown’ oomycete orders (Albuginales, Leptomitales, Peronosporales, Pythiales, Rhipidiales and Saprolegniales) for their sensitivity to the fungicide mandipropamid (MPD). Interestingly, those authors reported that only species belonging to the order of Peronosporales, of which *Hpa* is a member, were inhibited by this fungicide. Furthermore, Grenville‐Briggs *et al. *(2008) reported *CesA3* to be the most strongly expressed gene during mycelial growth of *Phytophthora*, *Saprolegnia* and *Pythium* species. Working with *P. infestans* using a protoplast transfection strategy, Grenville‐Briggs *et al. *(2008) used *in vitro*‐generated long dsRNA to silence *CesA* genes. From their studies, they concluded that silencing these genes leads to disruption of cell walls surrounding appressoria and the inability to form functional appressoria. Interestingly, they reported that *CesA3* is either less important or other *CesA* genes may be compensating for its loss of function (Grenville‐Briggs *et al.*, 2008).

Being an obligate plant pathogen, *Hpa* is not amenable to the genetic transformation and manipulation reported for *P. infestans* (Zheng *et al.*, 2014). Previously, we have tested several different methods, including electroporation, to obtain stable transgenic isolates and study gene functions. Although we observed marker gene expression in a few individual spores, we were unable to generate transgenic isolates or obtain uniformly transformed spore lines (N. Holton and M. Tör, unpublished data). Here, we used synthesized 25 nt sRNAs to silence the *CesA3* gene in *Hpa* by simply mixing the sRNAs with spores and inoculating cotyledons of seedlings or applying to cellophane strips. In inoculation experiments with antisense sRNAs, we showed a dose‐dependent reduction in sporulation on the inoculated cotyledons. This clearly indicates that (a) exogenously applied single‐stranded and ds sRNAs can be taken up by the spores without electroporation or any other transfection method, (b) the single‐stranded sRNAs somehow bind to the native *CesA3* RNA, forming a dsRNA and triggering the gene silencing machinery within the *Hpa* spores, (c) ds sRNA seems to be more effective in inhibiting infection than single‐stranded sRNA, (d) the silencing seems to be very effective, as *Hpa* is diploid and multinucleate, and (e) the *CesA3* gene is essential for pathogenicity of *Hpa*. Use of sense and antisense DNA oligonucleotides served as controls.

Trypan blue staining of seedlings inoculated with a spore suspension containing 20 µM antisense sRNAs revealed a lack of infection, indicating the inhibition of pathogen development at the germination stage; this was confirmed by the germination assays, which showed 100% inhibition of germination. Our results are in contrast to the findings of Grenville‐Briggs *et al.* (2008) where silencing *CesA* genes in *P. infestans* resulted in abnormal appressorium development rather than the inhibition of germination. This may well have been due to the redundancy factor as *P. infestans* has a larger genome than *Hpa* (Haas *et al.*, 2009).

Using the *Hpa*‐Cala2 genomic sequences (Woods‐Tör *et al.*, 2018), we found that the *CesA3* gene of Emoy2 has 99.97% identity to *CesA3* from Cala2 and the designed sRNA was gene‐specific. In addition, the inhibition of sporulation of the Cala2 isolate by the sRNA confirmed that the method is effective and that this gene is necessary for infection.

Ideally, we would design an sRNA molecule that could inhibit infection by several oomycete species. However, gene silencing relies on a conserved nucleotide sequence and, although the domains of CesA3 are conserved at the amino acid level across some of the important oomycete species, this is not so at the nucleotide level; unfortunately, there is not a conserved region of the nucleotide sequence that is long enough to design a common sRNA. Nevertheless, the method we developed here can easily be adapted to other oomycete species using newly designed gene‐specific sRNAs.

It is well known that all eukaryotic mRNA contains a cap structure, an N7‐methylated guanosine linked to the first nucleotide of the RNA (Ramanathan *et al.*, 2016). The cap has been reported to have several roles in cell viability, including promoting gene expression, mRNA stability and degradation, nuclear export of RNA and initiation of protein synthesis (Cowling, 2010). Recent studies also reported various cap structures in sRNAs in human cells (Abdelhamid *et al*., 2014), indicating that sRNAs can also go through a modification at their 5ʹ ends. Our inoculation studies clearly showed that the capping of sRNA was necessary for effective gene silencing. When *Hpa* spores are collected from infected seedlings, they are not derived from a sterile environment. Spore suspensions usually contain bacteria, tiny plant materials such as trichomes and other small substances. As the stability of exogenously applied sRNA in spore suspensions is very important, the cap structure of the sRNA may provide this required stability both outside and inside the spores.

In general, two classes of small non‐coding RNAs exist in plant cells: miRNAs (encoded by the genome) and siRNAs (derived from dsRNA produced by multiple sources) (Khraiwesh *et al*., 2012). In addition, the size of sRNAs in organisms can be different (Derbyshire *et al.*, 2018). Several studies revealed that, in plants and animals, each sRNA (acting as a guide) binds to an Argonaute family protein and a sequence‐specific gene silencing ribonucleoprotein (RNP) is formed by base pairing between the sRNA and its target mRNA; this is known as the RNA‐induced silencing complex (RISC) in miRNA and siRNA pathways (Budak and Akpinar, 2015; Vargason *et al.*, 2003; Wilson and Doudna, 2013).

In addition to the presence of a cap at the 5ʹ end of the sRNA, our results also showed the importance of the length of the sRNAs. We observed that 24 nt antisense sRNA did not inhibit infection, whereas it was completely inhibited by 25 or 30 nt antisense sRNAs. In a recent study, Åsman *et al. *(2016) co‐immunoprecipitated sRNAs with Argonaute proteins of the oomycete pathogen *P. infestans* and identified high enrichment of 24–26 nt sRNAs. In a similar study, Jia *et al. *(2017) sequenced sRNAs in another oomycete pathogen *Phytophthora parasitica* and reported that 25–26 nt sRNAs associate with efficient gene silencing in this pathogen. Although we do not know the exact reason of why 25 and 30 nt sRNAs silence *Hpa‐CesA3* but not 24 nt, it is tempting to speculate that the 24 nt sRNAs may not be binding to the *Hpa‐CesA3* transcript or may not be guiding the RISC complex to degrade *CesA3* mRNA.

Application of sRNA is more advantageous than a transgenic approach. Using this system, it should be possible to study pathogen development and pathogenicity in obligate pathogens. Till now, effectors from obligate oomycetes such as *Hpa* have been studied either by bombardment or via a bacterial delivery system to plants (Bailey *et al.*, 2011). An ideal method would be also to use reverse genetics to silence an effector gene within the pathogen and investigate whether this would alter pathogenicity. In addition, in some cases, effector genes in obligate pathogens are mapped to a locus where there are several genes within the interval. This system would allow rapid identification of the candidate gene. Using this simple method, we should now be able to study genes that are involved in pathogenicity and dissect different biological pathways of otherwise inaccessible obligate pathogens.

## Experimental Procedures

### Plant lines, pathogen isolates and propagation

*Hyaloperonospora arabidopsidis* isolates Emoy2 and Cala2 were maintained on *A. thaliana* Ws‐*eds1* (Parker *et al*., 1996). Preparation of inoculum for experiments was performed as described previously (Tör *et al.*, 2002). Sporulation was assessed 7 dpi, when the *Hpa* life cycle had been completed. To quantify sporulation, ten infected seedlings from each replicate were taken and placed into an Eppendorf tube containing 250 µl H_2_O. Samples were vortexed and conidiospores were counted using a haemocytometer.

### sRNA and DNA oligonucleotide synthesis

The *Hpa‐CesA3* gene (*HpaG810051*) was used as the target gene in this method. Sense and antisense sRNAs designed at various lengths were Hpa_CesA3_RNA_AS_24 5ʹ‐GCCGCAUCGCACGUACCUCAGUAC‐3', Hpa_CesA3_RNA_AS_25 5'‐GCCGCAUCGCACGUACCUCAGUACG‐3', Hpa_CesA3_RNA_S_25, 5'‐CGUACUGAGGUACGUGCGAUGCGGC‐3' and Hpa_CesA3_RNA_S_30 5ʹ‐GUCGUACUGAGGUACGUGCGAUGCGGCACU‐3ʹ. Hpa_CesA3_RNA_AS_30 5'‐AGUGCCGCAUCGCACGUACCUCAGUACGAC‐3' and double‐stranded sRNA were generated by mixing equal volume of Hpa_CesA3_RNA_S_30 and Hpa_CesA3_RNA_AS_30 oligos, heating at 95 ˚C for 5 min and allowing to anneal at room temperature for 20 min.

Similarly, sense and antisense DNA oligonucleotides were also designed to the same region. These were Hpa_CesA3_DNA_S_25 5ʹ‐GCCGCATCGCACGTACCTCAGTACG‐3ʹ and Hpa_CesA3_DNA_AS_25 5ʹ‐CGTACTGAGGTACGTGCGATGCGGC‐3ʹ. These were obtained as synthesized deoxyribonucleotides or ribonucleotides from Sigma (Gillingham, UK) or Eurofin (Ebersberg, Germany).

### Application of sRNAs to pathogen spores and plant inoculations

*Hpa* spores were collected from infected *A. thaliana* Ws‐*eds1* seedlings, washed twice in sterile distilled water and the spore concentration was adjusted to 5 × 10^4^/mL using a haemocytometer. sRNAs were added to the spore suspension at a final concentration of 5, 10 and 20 µM and spores were subsequently drop inoculated onto 7‐day‐old seedlings. As a control, seedlings were also inoculated with spores in the same way with 20 µM DNA oligonucleotides or without sRNAs or DNA oligonucleotides. Seedlings were inspected from 3 dpi for sporulation. Sporulation was quantified as described above.

### Spore germination assays

MS medium (Murashige and Skoog, 1962) was prepared with 4.3 g/L MS basal salt mixture powder (Sigma, M5524), agar (1.5%) (Sigma, A1296), sucrose (10 g/L) (Sigma, 84100), and distilled water. MS powder was dissolved in sterile distilled H_2_O, the pH was adjusted to 5.7 using 1 M NaOH/HCl, and agar and sugar were added. The medium was sterilized by autoclaving at 15 psi and 121 °C for 15 min. Approximately 20 mL of the medium was aliquoted into each sterile Petri dish in a laminar airflow unit.

Cellophane strips, 1.5 cm in length, were cut from plain transparent florists' cellophane and autoclaved in distilled water. After autoclaving, cellophane strips were placed onto the MS medium in Petri dishes under a laminar airflow and dishes were kept in the fridge for long‐term storage.

*Hpa* spores were collected, washed twice in sterile distilled water and the spore concentration was adjusted to 5 × 10^4^ spores/mL using a haemocytometer. Approximately 10 µL spore suspension, with 0 or 20 µM antisense sRNA, was dropped onto each piece of cellophane. Plates were incubated with a 12 h light/12 h dark regime at 16 °C. Spores were examined under a light microscope 48 h after incubation and germinated spores were counted.

### Staining plant tissues

Seedlings of infected and non‐inoculated controls were stained with a solution of phenol, lactic acid, glycerol and water (1:1:1:1) supplemented with 1 mg/mL trypan blue, decolorized in chloral hydrate and visualized under a compound microscope as described in Woods‐Tör *et al.* (2018).

### Statistical analysis

For statistical analysis, paired Student’s *t*‐tests were performed on data obtained from plant infection assays.

### Bioinformatics

IICB Genomics and Transcriptomics Resources (http://eumicrobedb.org) and the EnsemblProtist (http://protists.ensembl.org) database were used for information on *Hpa.* Web servers including InterPro (Quevillon *et al.*, 2005) (http://www.ebi.ac.uk/interpro/) and Pfam (Punta *et al.*, 2012) ((http://pfam.wustl.edu/) were used for the analysis of *Hpa*‐CesA3. BLAST (Altschul *et al.*, 1997) was used to perform similarity‐search of nucleotide and amino acid sequences of *Hpa‐CesA3* against oomycete and *Arabidopsis* sequences. Primer design was performed using Geneious v. 10.0 (Kearse *et al.*, 2012).

## Author Contributions

M.T. and Y.H. planned and designed the research. Ö.B., O.T., C.N. and M.T. conducted the laboratory work. M.T., Y.H. and H.B. analysed and interpreted the data and wrote the manuscript.

## Conflict of Interest

The authors declare that there is no conflict of interest.

## Supporting information


**Fig. S1** Comparison of CesA3 amino acid sequences from different oomycete pathogens. Amino acid sequences of CesA3 proteins from *Hyaloperonospora arabidopsidis* (*Hpa*, M4BU64), *Albugo candida* (AFB77612), *Albugo laibachii* (CCA23182), *Bremia lactucae* (AFB20351), *Phytophthora capsici* (AFB20353), *Phytophthora infestans* (ABP9690), *Plasmopara viticola* (ADD84672) were aligned using Geneious v. 10. Black or dark grey boxes with white letters indicate identity or similarity to *Hpa*‐CesA3, respectively.

**Fig. S2** Comparison of *CesA3* nucleotide sequences from different oomycete pathogens. Nucleotide sequences of CesA3 gene from *Hyaloperonospora arabidopsidis* (*Hpa*), *Albugo candida*, *Albugo laibachii*, *Bremia lactucae*, *Phytophthora capsici*, *Phytophthora infestans*, *Plasmopara viticola* were aligned using Geneious v. 10. Black or dark grey boxes with white letters indicate identity or similarity to *Hpa‐CesA3*, respectively.

**Fig. S3** Expression pattern of *Hpa‐CesA3*. Expression levels were represented as TPM (tags per million) of total reads mapped to *Hyaloperonospora arabidopsidis* genome. Data was acquired from Asai *et al*. (2018). Cs, conidiospore, dpi, days post‐inoculation.

**Fig. S4** Sense and antisense DNA oligonucleotides do not inhibit sporulation. *Arabidopsis* seedlings were drop inoculated with *Hpa*‐Emoy2 spores containing 20 µM sense or antisense DNA oligonucleotides. Inoculated 10 seedlings from each sample were collected 7 days post‐inoculation and placed in 250 µL H_2_O. The number of spores was counted using a heamocytometer. Averages and standard errors of three replicates are shown. Experiment was repeated three times with similar results.

**Fig. S5** Nucleotide sequence alignment of *Hpa‐CesA3* from Emoy2 and Cala2 isolates. Sequences were aligned using Geneious v. 10. Black or dark grey boxes with white letters indicate identity or similarity to *Hpa‐CesA3* from Emoy2, respectively. sRNA indicates the sequences where sRNAs were designed from.

**Table S1** Spore germination assay using antisense sRNA.

**Table S2** Number of spores detected in seedlings inoculated with dsRNA.

## Data Availability

The data that support the findings of this study are available from the corresponding author on reasonable request.
