# Supplementary figures and images for: Small RNA inhibits infection by downy mildew pathogen Hyaloperonospora arabidopsidis
Source: Mol Plant Pathol. 2019 Sep 26;20(11):1523–34. doi: 10.1111/mpp.12863 (PMC6804343; doi:10.1111/mpp.12863)

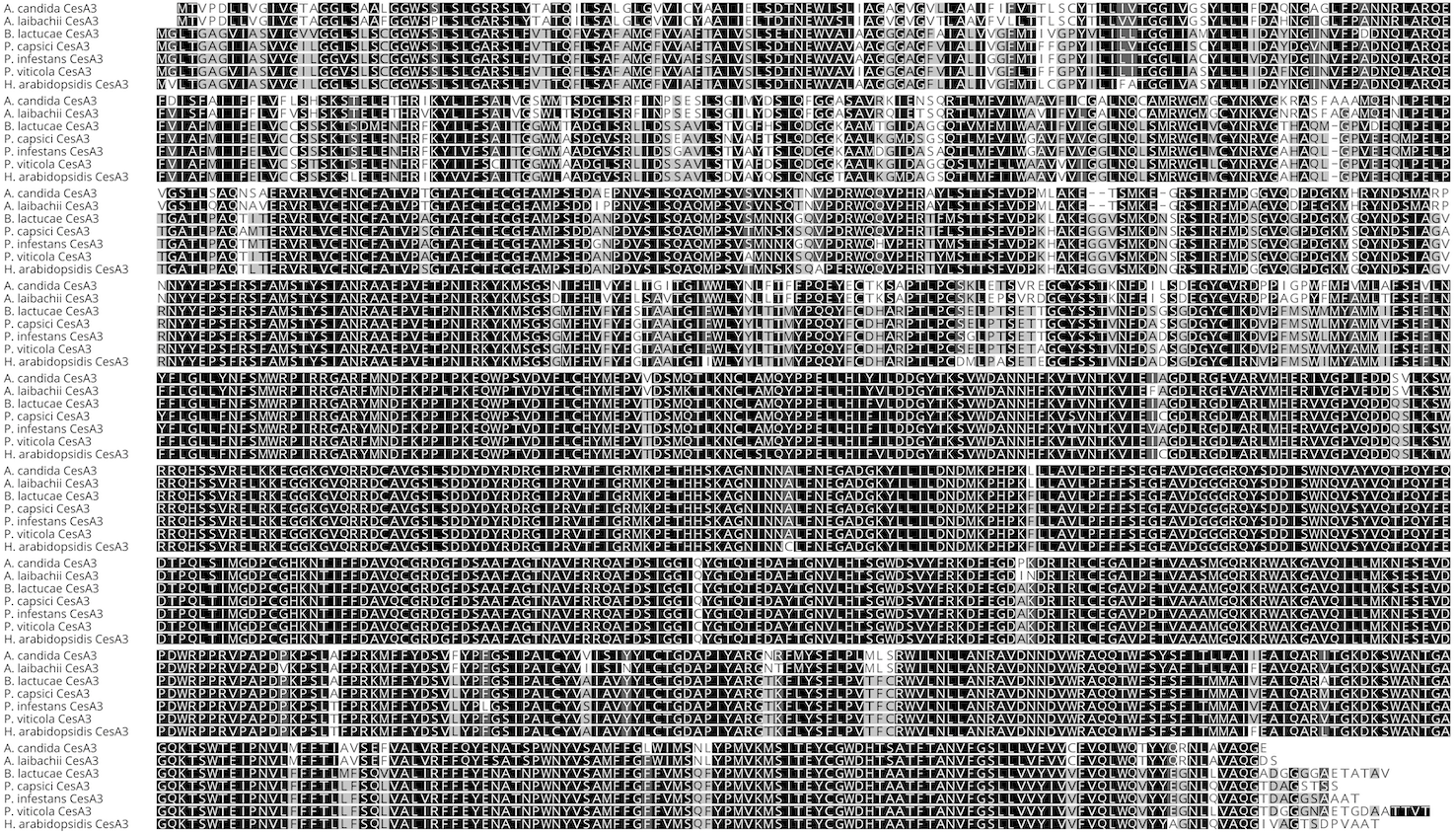

Supplement: Supplementary file 1 — Fig. S1 Comparison of CesA3 amino acid sequences from different oomycete pathogens. Amino acid sequences of CesA3 proteins from Hyaloperonospora arabidopsidis (Hpa, M4BU64), Albugo candida (AFB77612), Albugo laibachii (CCA23182), Bremia lactucae (AFB20351), Phytophthora capsici (AFB20353), Phytophthora infestans (ABP9690), Plasmopara viticola (ADD84672) were aligned using Geneious v. 10. Black or dark grey boxes with white letters indicate identity or similarity to Hpa‐CesA3, respectively. [file MPP-20-1523-s001.tiff]

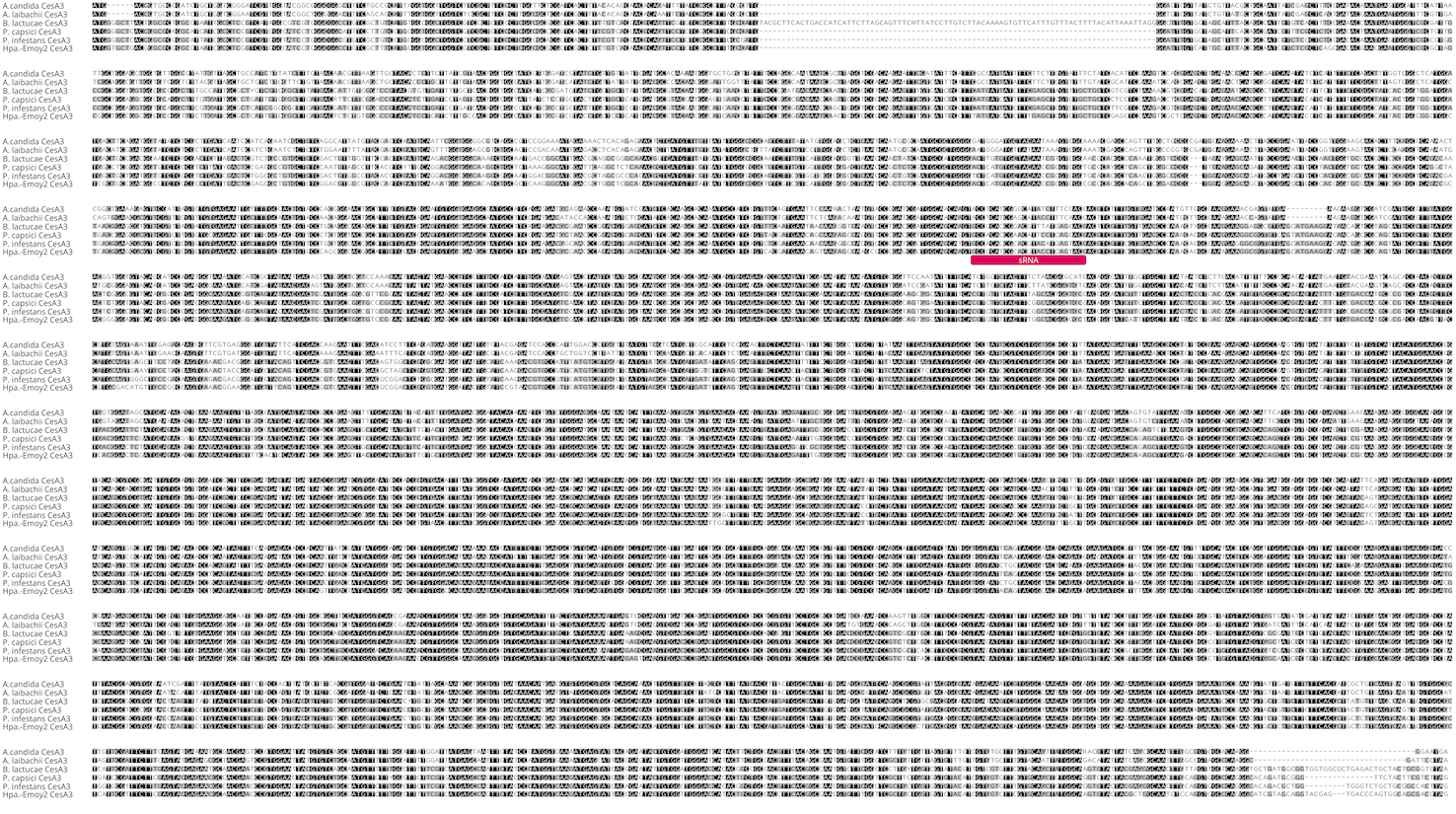

Supplement: Supplementary file 2 — Fig. S2 Comparison of CesA3 nucleotide sequences from different oomycete pathogens. Nucleotide sequences of CesA3 gene from Hyaloperonospora arabidopsidis (Hpa), Albugo candida, Albugo laibachii, Bremia lactucae, Phytophthora capsici, Phytophthora infestans, Plasmopara viticola were aligned using Geneious v. 10. Black or dark grey boxes with white letters indicate identity or similarity to Hpa‐CesA3, respectively. [file MPP-20-1523-s002.tiff]

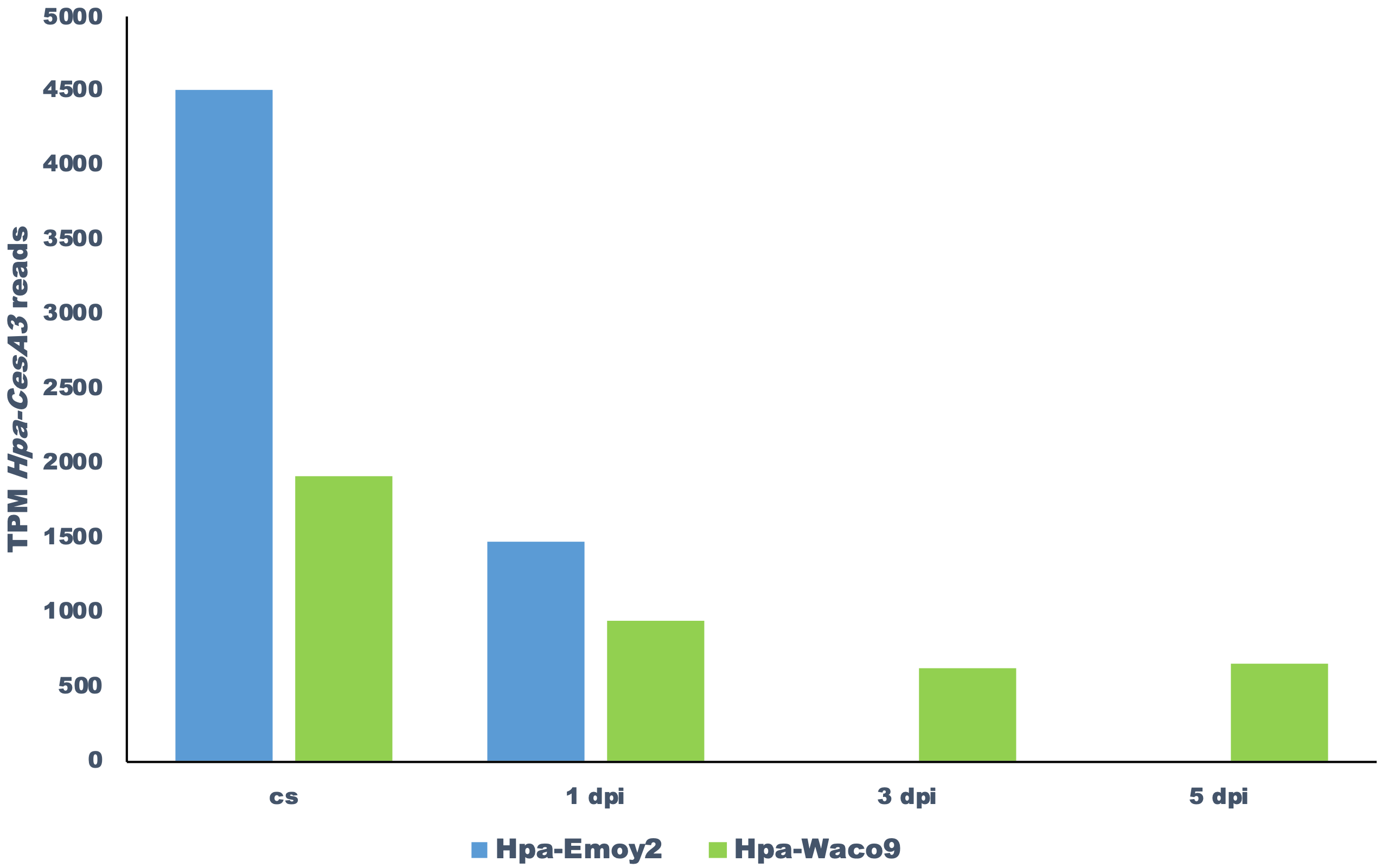

Supplement: Supplementary file 3 — Fig. S3 Expression pattern of Hpa‐CesA3. Expression levels were represented as TPM (tags per million) of total reads mapped to Hyaloperonospora arabidopsidis genome. Data was acquired from Asai et al. (2018). Cs, conidiospore, dpi, days post‐inoculation. [file MPP-20-1523-s003.tiff]

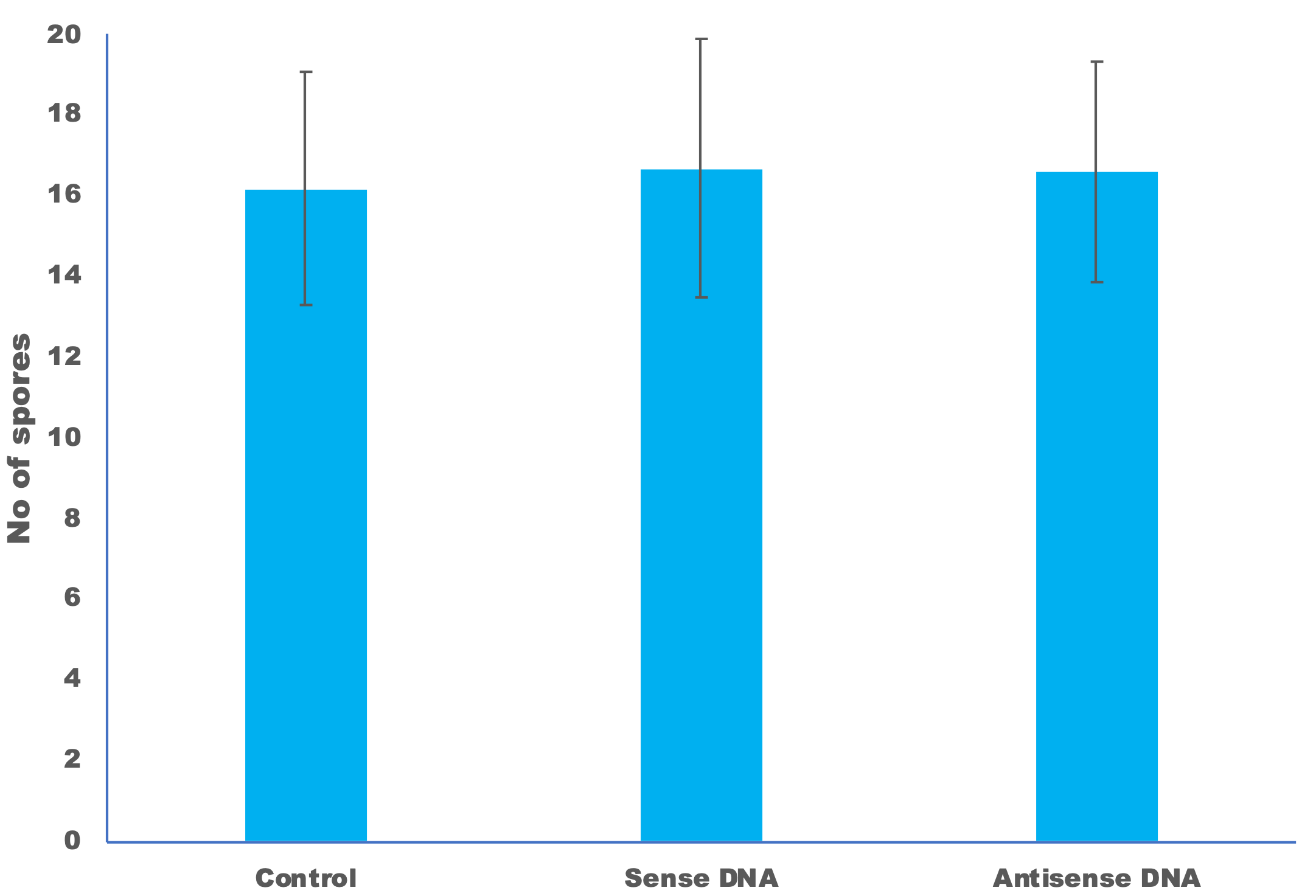

Supplement: Supplementary file 4 — Fig. S4 Sense and antisense DNA oligonucleotides do not inhibit sporulation. Arabidopsis seedlings were drop inoculated with Hpa‐Emoy2 spores containing 20 µM sense or antisense DNA oligonucleotides. Inoculated 10 seedlings from each sample were collected 7 days post‐inoculation and placed in 250 µL H2O. The number of spores was counted using a heamocytometer. Averages and standard errors of three replicates are shown. Experiment was repeated three times with similar results. [file MPP-20-1523-s004.tiff]

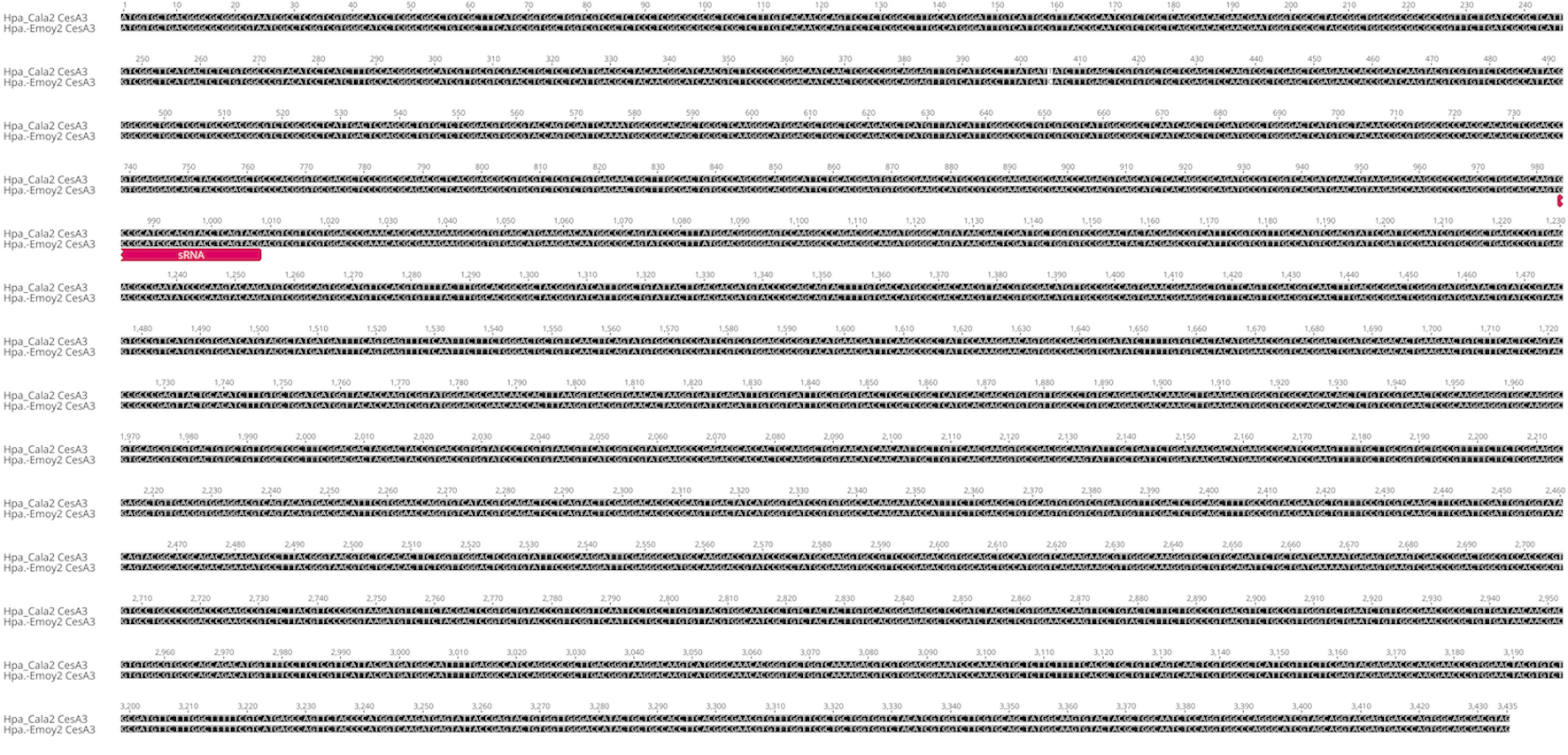

Supplement: Supplementary file 5 — Fig. S5 Nucleotide sequence alignment of Hpa‐CesA3 from Emoy2 and Cala2 isolates. Sequences were aligned using Geneious v. 10. Black or dark grey boxes with white letters indicate identity or similarity to Hpa‐CesA3 from Emoy2, respectively. sRNA indicates the sequences where sRNAs were designed from. [file MPP-20-1523-s005.tiff]
